# *IL13 *gene polymorphisms modify the effect of exposure to tobacco smoke on persistent wheeze and asthma in childhood, a longitudinal study

**DOI:** 10.1186/1465-9921-9-2

**Published:** 2008-01-10

**Authors:** Alireza Sadeghnejad, Wilfried Karmaus, S Hasan Arshad, Ramesh Kurukulaaratchy, Marianne Huebner, Susan Ewart

**Affiliations:** 1 Center for Human Genomics, Wake Forest University School of Medicine, Winston-Salem, North Carolina, USA; 2 Department of Epidemiology and Biostatistics, University of South Carolina, Columbia, South Carolina, USA; 3 The David Hide Asthma & Allergy Research Centre, St. Mary's Hospital, Isle of Wight, UK; 4 IIR Research Division, School of Medicine, University of Southampton, Southampton, UK; 5 Department of Statistics and Probability, Michigan State University, East Lansing, Michigan, USA; 6 Department of Large Animal Clinical Sciences, Michigan State University, East Lansing, Michigan, USA

## Abstract

**Background:**

Tobacco smoke and genetic susceptibility are risk factors for asthma and wheezing. The aim of this study was to investigate whether there is a combined effect of interleukin-13 gene (*IL13*) polymorphisms and tobacco smoke on persistent childhood wheezing and asthma.

**Methods:**

In the Isle of Wight birth cohort (UK, 1989–1999), five *IL13 *single nucleotide polymorphisms (SNPs): rs1800925 (-1112C/T), rs2066960, rs1295686, rs20541 (R130Q) and rs1295685 were genotyped. Parents were asked whether their children had wheezed in the last 12 months at ages 1, 2, 4 and 10 years. Children who reported wheeze in the first 4 years of life and also had wheezing at age 10 were classified as *early-onset persistent *wheeze phenotype; *non-wheezers *never wheezed up to age 10. Persistent asthma was defined as having a diagnosis of asthma both during the first four years of life and at age 10. Logistic regression methods were used to analyze data on 791 children with complete information. Potential confounders were gender, birth weight, duration of breast feeding, and household cat or dog present during pregnancy.

**Results:**

Maternal smoking during pregnancy was associated with *early-onset persistent *wheeze (OR 2.93, *p *< 0.0001); polymorphisms in *IL13 *were not (OR 1.15, *p *= 0.60 for the common haplotype pair). However, the effect of maternal smoking during pregnancy was stronger in children with the common *IL13 *haplotype pair compared to those without it (OR 5.58 and OR 1.29, respectively; *p *for interaction = 0.014). Single SNP analysis revealed a similar statistical significance for rs20541 (*p *for interaction = 0.02). Comparable results were observed for persistent childhood asthma (*p *for interaction = 0.03).

**Conclusion:**

This is the first report that shows a combined effect of *in utero *exposure to smoking and *IL13 *on asthma phenotypes in childhood. The results emphasize that genetic studies need to take environmental exposures into account, since they may explain contradictory findings.

## Background

It has been suggested that the increased prevalence of asthma-related phenotypes over the last three decades is due to exposure to environmental factors [1]. Among such exposures, environmental tobacco smoke is regarded as an important risk factor for asthma-related phenotypes [2,3]. In particular, an effect of maternal smoking during pregnancy on the development of asthma in offspring has been proposed [2,4-7]. Parental smoking has also been reported to be associated with markers of atopy, such as serum immunoglobulin E (IgE) levels [8]. Interleukin-13 (IL-13) is an important cytokine involved in the IgE pathway and pathogenesis of asthma [9]. Pulmonary expression of *IL13 *is reported to produce inflammation, mucus hypersecretion, subepithelial fibrosis and eotaxin production [10]. It has been reported that exposure to tobacco smoke during pregnancy was associated with a significantly higher production of IL-13 [11,12]. These observations suggest that part of the effect of exposure to tobacco on asthma-related phenotypes might be through cytokines.

Researchers have speculated that individuals may vary in genetic susceptibility to the physiologic and cellular response to cigarette smoke exposure and a few studies have examined gene-smoking interactions for asthma [13-16]. Wang *et al*. observed that an interaction between maternal smoking and the β_2 _adrenergic receptor gene was associated with asthma in offspring [15]. Kabesch *et al*. demonstrated an interaction between smoking and glutathione S transferase deficiency on asthma [16]. In two genome-wide screens, interactions between passive tobacco exposure and chromosome 5q, the region in which the IL-13 gene (*IL13*) is located, have been reported for asthma phenotypes [13,14]. Recently we reported an interaction of smoking and the *IL13 *single nucleotide polymorphism (SNP) rs1800925 related to reduced lung function in long term tobacco smokers [17].

Compared to wild type IL-13, a non-synonymous polymorphism in the *IL13 *gene, rs20541 (+2044 G/A, R130Q), has been shown to be associated with more active IL-13 [18]. It has also been reported that the rs1800925 (-1112C/T) polymorphism resulted in enhanced promoter activity [19]. In spite of the importance of IL-13 in asthma [9,11,12], some studies failed to show an association between *IL13 *polymorphisms and asthma phenotypes [20,21], possibly because of different prevalence of environmental risk factors such as tobacco smoke exposure.

Because of the high prevalence (25–30%) of smoking among women of reproductive age [22] there is a need to investigate its possible role in the development of asthma-related phenotypes. In prior assessments of the Isle of Wight birth cohort study, we have shown that children with *early-onset persistent *wheeze have the highest amount of morbidity in association with atopic sensitization, hospital admission and steroid therapy for asthma [23] To our knowledge there is no investigation as to whether the adverse effect of maternal smoking on asthma may result from an interaction between exposure to tobacco smoke and cytokine regulating genes. In the current work we test the hypothesis that an interaction between maternal smoking during pregnancy and the *IL13 *gene is associated with increased risk of *early-onset persistent *wheeze and persistent childhood asthma in the Isle of Wight birth cohort.

## Methods

### Population and data

The local Research Ethics Committee approved the study of children born and enrolled (n = 1,456) between January 1, 1989, and February 28, 1990, on the Isle of Wight, United Kingdom. Written consent was obtained and children were followed up at the ages of 1, 2, 4 and 10 years. The island is close to the British mainland, semi-rural, with no heavy industry. The population is 99% Caucasian.

At birth, weight was obtained from hospital records and parents were asked about their age and exposures during pregnancy. At subsequent follow-ups, a questionnaire was administered seeking information on exposure to tobacco smoke, breast feeding, and wheezing in the cohort children.

### Exposure to tobacco smoke

Information on tobacco smoking by mothers (during pregnancy and later), fathers, or any other individual inside the home was recorded at recruitment and updated at each follow-up. Exposures to environmental tobacco smoke (ETS) in the household and maternal smoking during pregnancy were combined and classified into three groups. When mothers did not smoke during pregnancy and there was no exposure to household ETS in children up to the age of 10, children were categorized as "ETS-0". When mothers did not smoke during pregnancy, but household members smoked within the home at some point up to the child's age of 10 years, the exposure status was categorized as "ETS-1". When mothers smoked during pregnancy and the children were also exposed to household ETS at some point up to the age of 10, the exposure was categorized as "ETS-2". None of the children had mothers who smoked during pregnancy but no exposure to household tobacco smoke after birth.

### Definition of wheeze phenotypes and persistent childhood asthma

At each of the four follow-ups at ages 1, 2, 4, and 10 years, "current wheeze" was recorded if wheezing occurred on at least one occasion in the previous 12 months. Parents were also asked whether the child had any wheeze episode between ages 4 and 10. Children were then categorized into wheezing phenotypes as follows: *non-wheezers *never wheezed in the first decade of life, *early-onset persistent *wheezers had wheezed in the first 4 years of life and still wheezed at age 10, while *late-onset persistent *wheezers had wheezing onset after 4 years of age and still wheezed at age 10. *Early transient *wheezers began wheezing in the first 4 years but stopped at least 12 months before age 10. Details on wheeze phenotypes from 1 to 10 years of this cohort have been reported previously [23].

Asthma was based on investigator diagnosis of asthma and has been described in previous reports of this cohort [24]. Persistent childhood asthma was defined as having asthma diagnosis at age 10 in addition of at least one asthma diagnosis in the first four years of life, the control group included children with no asthma diagnosis at any time point, case-cohort analysis.

### DNA isolation and *IL13 *genotyping

Anticoagulated whole blood samples were obtained at the 10-year interview and stored frozen (n = 921). Genomic DNA was isolated from these samples using QIAamp DNA Blood Kits (Qiagen, Valencia, CA) or the ABI PRISM™ 6100 Nucleic Acid PrepStation (Applied Biosystems, Foster City, CA). Polymorphisms in the *IL13 *gene were examined using the SNPper and Applied Biosystems databases [25]. Genotyping was conducted by fluorogenic 5' nuclease chemistry PCR using Assays on Demands kits cycled on a 7900HT Sequence Detection System (Applied Biosystems, Foster City, CA), or biotin-streptavidin-based pyrosequencing performed on PSQ-96 instrumentation (Biotage AB, Uppsala, Sweden).

Five SNPs from the *IL13 *gene were used in this study: rs1800925 in the 5' promoter region, rs2066960 in intron 1, rs1295686 in intron 3 at the intron/exon boundary, rs20541 in exon 4 and rs1295685 in the 3' untranslated region (UTR) of exon 4. The rs20541 is a coding variant with the common allele (G) coding for arginine and the minor allele (A) encoding glutamine at amino acid 144. Because *IL13 *is a small gene (2.9 kb), only a few SNPs were needed for a reasonable assessment of genetic associations. The SNPs in this report build upon the haplotype tagging (ht)-SNPs indicated by Tarazona-Santos and Tishkoff in a European population [26] and they include those most commonly examined for association with asthma and related phenotypes in other previous studies [27-31].

### Statistical analysis

Each SNP was tested for Hardy-Weinberg equilibrium using Haploview 3.2 software [32]. Estimates of linkage disequilibrium (LD) between SNPs were calculated using *D*' and *r*^2 ^[33]. PHASE 2.0.2 was employed to build the most likely pair of haplotypes (diplotypes) and their probability for each child [34], using all five SNPs. In the analysis of individual SNPs and haplotype pairs, genotypes with low frequencies were combined [35,36].

Using SAS/STAT^® ^version 9.1, statistical analysis was performed on the data only from children who had complete information on *IL13 *genotypes, exposure to tobacco smoke and wheeze phenotypes. Chi-square tests were used to compare the sample used in the analysis with that which was followed up at age 10. To investigate main effects and interactions between smoke exposures and genetic polymorphisms for the wheeze phenotypes, a multinomial logistic regression analysis (PROC GLIMMIX, SAS) was conducted. For analysis of persistent asthma simple logistic regression (PROC LOGISTICS, SAS) was used.

To evaluate the associations between wheeze phenotypes and other variables of interest, *non-wheezers *were used as the control group. Persistent childhood asthma was treated as a dichotomous variable (yes, no). Confounders were chosen based on their importance in studying wheeze phenotypes in a prior report of the Isle of Wight cohort [37]. Potential confounders (table 1) were: gender, low birth weight (< 2,500 grams versus ≥ 2,500 grams), breast feeding (< three months and ≥ three months), household cat present during pregnancy (yes, no) and household dog present during pregnancy (yes, no).

**Table 1:** Comparison of children with a follow-up at age 10 and the subset used in the analysis

		Numbers at age 10 (%)	Numbers used in the analysis (%)	χ^2 ^*p *value
**Variable**	**Total**	1,373	791	

Wheeze Phenotype	Early-onset persistent*	139 (12.7)	105 (13.3)	0.60
	*Late-onset persistent*†	81 (7.4)	70 (8.9)	
	*Early transient*‡	259 (23.6)	190 (23.9)	
	*Non-wheezer*§	617 (56.3)	426 (53.9)	
	Missing	277	0	
Birth weight	≥2500 grams	1287 (96.3)	736 (96.0)	0.75
	<2500 grams	49 (3.7)	31 (4.0)	
	Missing	37	24	
Exposure to tobacco smoke	ETS-0	617 (45.2)	381 (48.2)	0.22
	ETS-1	430 (31.5)	249 (31.5)	
	ETS-2	319 (23.3)	161 (20.3)	
	Missing	7	0	
Gender	Girl Boy	676 (49.2)	388 (49.3)	0.98
		697 (50.8)	399 (50.7)	
Breast fed	≥3 months	470 (41.7)	322 (42.8)	0.67
	<3 months	657 (58.3)	430 (57.2)	
	Missing	246	39	
Household cat during pregnancy	Yes	453 (33.2)	269 (34.0)	0.68
	No	912 (66.8)	521 (66.0)	
	Missing	8	1	
Household dog during pregnancy	Yes	395 (28.9)	240 (30.4)	0.48
	No	970 (71.1)	550 (69.6)	
	Missing	8	1	

* Onset of wheeze in the first 4 years, still present at age 10.

† Onset of wheeze after age 4 years, still present at age 10.

‡ Wheeze only in the first 4 years.

§ No wheeze up to age 10.

ETS-0, mothers did not smoke during pregnancy and children not exposed to household ETS; ETS-1, mothers did not smoke during pregnancy, but children were exposed to household ETS; ETS-2, mothers smoked during pregnancy and children were exposed to household ETS

## Results

Data on *IL13 *genotypes, exposure to tobacco smoke and wheeze phenotypes were available for 791 children. The percentages of children who wheezed and those who had ETS-0 were higher in the sample used in the analyses compared to all those who were followed up to age 10 (table 1). More than half of the children were exposed to tobacco smoke up to age 10 (23.3% during and after pregnancy; 31.5% after pregnancy, table 1).

**Figure 1:**
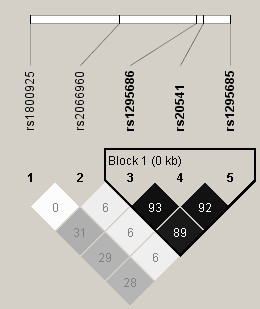
**Using the Haploview program [32], three single nucleotide polymorphisms of the interleukin 13 gene appeared to be in a block**. *r*^2^, a pair wise linkage disequilibrium determinant [33], is shown.

For *IL13 *genotypes, all five SNPs were in Hardy-Weinberg equilibrium and three of the SNPs (rs1295686, rs20541 and rs1295685) demonstrated linkage disequilibrium based on *D*' (≥ 0.95) and *r*^2 ^(≥ 0.89, Figure 1). Therefore, haplotypes were inferred using rs20541 from this block and the other two SNPs (rs1800925 and rs2066960). Due to the limited number of children homozygous for minor alleles at all SNPs (less than 5%, table 2), minor allele homozygous and heterozygous genotypes were grouped together. Among haplotype pairs, CCG/CCG (for SNPs ordered rs1800925, rs2066960, rs20541) had the highest frequency (0.48, table 3). The estimated probability for CCG/CCG as the best pair was 1.0 in 99% of children and at least 0.89 in the rest (results not shown). All other haplotype pairs had frequencies between 0.019–0.155. In addition, there were probabilities as low as 0.5 that were identified as the best pair for haplotype pairs other than CCG/CCG. Thus, haplotype pairs other than CCG/CCG were combined [35,36].

**Table 2:** Genotypes for *IL13 *single nucleotide polymorphisms (SNPs)

SNP	Position (bp)	Location	Genotype	Frequency (%)
rs1800925 (-1112C/T)	132,020,708	Promoter	CC	577 (63.6)
			CT	295 (32.5)
			TT	35 (3.9)
			Total	907 (100.0)
rs2066960	132,022,334	Intron 1	CC	729 (81.5)
			AC	157 (17.6)
			AA	8 (0.9)
			Total	894 (100.0)
rs1295686	132,023,742	Intron 3	CC	483 (64.6)
			CT	240 (32.1)
			TT	25 (3.3)
			Total	748 (100.0)
rs20541 (R130Q)	132,023,863	Exon 4	GG	583 (64.4)
			GA	291 (32.1)
			AA	32 (3.5)
			Total	906 (100.0)
rs1295685	132,024,344	Exon 4	GG	584 (64.5)
			GA	280 (30.9)
			AA	41 (4.5)
			Total	905 (100.0)

Gene annotation is based on SNPper [25]

*IL13*: interleukin-13 gene; n, number of samples used in genetic studies; bp, basepairs.

**Table 3:** Frequency of haplotypes and haplotype pairs for five single nucleotide polymorphisms (rs1800925, rs2066960, rs20541) of *IL13*, inferred from genotype data of 912 children

		Frequency (SE)
Haplotype	CCG	0.687 (0.003)
	TCA	0.105 (0.003)
	TCG	0.067 (0.002)
	CCA	0.043 (0.002)
	CAG	0.040 (0.003)
	CAA	0.027 (0.002)
	TAA	0.020 (0.003)
	TAG	0.009 (0.002)
Haplotype pair(s)	CCG/CCG	0.478
	CCG/minor haplotypes*	0.451
	minor haplotypes/minor haplotypes†	0.071

PHASE 2.0.2. was used for this analysis [34].

* This group consists of, with a frequency of:

CCG/TCA, 0.155; CCG/TCG, 0.088; CCG/CCA, 0.055; CCG/CAG, 0.054;

CCG/TAA, 0.043; CCG/CAA, 0.038; CCG/TAG, 0.019;

† This group consists of, with a frequency of:

TCG/TCA, 0.020; CCA/TCA, 0.012; other haplotypes, < 0.010

*IL13*: interleukin-13

To determine the associations of *IL13*, exposure to tobacco smoke and their combination on wheeze phenotypes, we conducted multinomial logistic regression. Crude analysis showed that, with the exception of rs2066960 with *early-transient *wheeze, genotypes of individual SNPs or haplotype pairs were not associated with *early-onset persistent *wheeze (table 4, reference:*non-wheezers*) or other wheeze phenotypes; maternal smoking during pregnancy (ETS-2) was associated with *early-onset persistent *wheeze (*p *< 0.0001, table 4).

**Table 4:** Unadjusted Odds Ratios, multinomial logistic regression, for covariates on wheeze phenotypes

	*Early-transient*	*Early-onset persistent*	*Late-onset*
	
Covariate	OR, p-value	OR, p-value	OR, p-value
rs1800925, CT-TT vs CC	0.99, 0.971	0.98, 0.928	0.91, 0.743
rs2066960, CA-AA vs CC	1.61, 0.032	1.36, 0.273	1.24, 0.524
rs1295686, CT-TT vs CC	1.02, 0.922	1.12. 0.642	1.02, 0.937
rs20541, GA-AA vs GG	1.17, 0.393	1.12, 0.614	1.55, 0.096
rs1295685, GG vs GA-AA	1.16, 0.411	1.15, 0.535	1.51, 0.116
others vs CCG/CCG	1.10, 0.592	0.93, 0.729	1.10, 0.709
ETS-2 vs ETS-0	1.70, 0.022	2.93, <0.0001	0.57, 0.173
ETS-1 vs ETS-0	1.57, 0.023	1.86, 0.018	0.61, 0.861

‡ The reference was the group who never report wheeze up to age 10 (n = 426).

CCG/CCG is the most common haplotype pair constructed based on rs1800925, rs2066960 and rs20541, using PHASE 2.0.2 software [34]. ETS-0, mothers did not smoke during pregnancy and children not exposed to household ETS; ETS-1, mothers did not smoke during pregnancy, but children were exposed to household ETS; ETS-2, mothers smoked during pregnancy and children were exposed to household ETS

**Figure 2:**
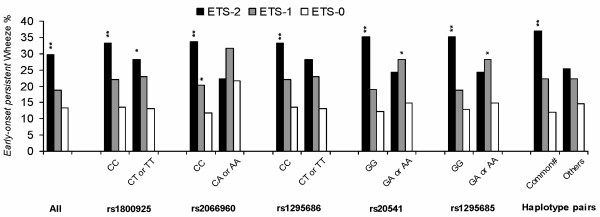
**The vertical axis is the percentage of *early-onset persistent *wheezers divided by the sum of *early-onset persistent *wheezers and *non-wheezers *(n = 791)**. For each genotype strata, reference was ETS-0. *, *p *value < 0.05; **, *p *value < 0.001. ETS-0, mothers did not smoke during pregnancy and children were not exposed to household ETS; ETS-1, mothers did not smoke during pregnancy, but children were exposed to household ETS; ETS-2, mothers smoked during pregnancy and children were exposed to household ETS. SNP, single nucleotide polymorphism; rs1800925, rs2066960, rs1295686, rs20541 and rs1295686 are five SNPs within the interleukin13 gene. SNPs rs1295686, rs20541, and rs1295686 were in a block. Therefore haplotype analysis was based on SNPs rs1800925, rs2066960, and rs20541. # The most common haplotype pair, CCG/CCG, constructed based on SNPs rs1800925, rs2066960, and rs20541, using PHASE 2.0.2 software [34].

In a stratified analysis, the combined effect of *IL13 *genotypes and exposure to tobacco smoke on *early-onset persistent *wheeze was assessed. In the strata with common (homozygous major allele)*IL13 *genotypes, the effects of maternal smoking during pregnancy on *early-onset persistent *wheeze were significant (figure 2). In particular, figure 2 shows for the five SNPs and for the *IL13 *haplotype that common genotypes were not related to a higher prevalence of wheezing in non-smoke exposed children (white bars). However, under the condition of prenatal smoke exposure, children with common genotypes showed a statistically increased risk of *early-onset persistent* wheezing (black bars). This combined effect was not found for children who had minor homozygous or heterozygous genotypes.

Table 5 shows a model to evaluate the statistical interaction of CCG/CCG and smoking. For *early-onset persistent *wheeze, the interaction term between ETS-2 and CCG/CCG showed statistical significance (*p *< 0.014, table 5). For this combined effect the two different ORs (OR = 2.59 and OR = 5.89) are calculated based on two different scenarios. In the first case (OR = 2.59), children exposed to maternal smoking during pregnancy are the reference and the effect of the gene is added to this equation, thus, given this reference, the effect of maternal smoking during pregnancy is eliminated from the equation. In the second case (OR = 5.89), children with the CCG/CCG haplotype pair are the reference and maternal smoking during pregnancy is introduced to this setting. In addition to the haplotype analysis, single SNP analysis revealed a significant interaction term between rs20451 (GG versus GA+AA) and ETS-2 on *early-onset persistent *wheeze (*p *for interaction < 0.020, data not shown).

**Table 5:** Adjusted estimates, multinomial logistic regression model, for the effect of smoke exposure and the major haplotype pair (CCG/CCG)† of *IL13 *gene on wheeze phenotypes

*Variable*	Function‡	estimate	p value	Odds ratio & 95% CI		
Boy	1	0.52	0.006	1.69	1.16	2.49
	**2**	**0.64**	**0.009**	**1.90**	**1.18**	**3.06**
	*3*	*0.29*	*0.275*	*1.33*	*0.80*	*2.23*
Birth weight < 2500 g	1	-1.11	0.012	0.33	0.14	0.78
	**2**	**-0.10**	**0.887**	**0.91**	**0.24**	**3.49**
	*3*	*-0.14*	*0.863*	*0.87*	*0.19*	*4.09*
Breast feeding < 3 months	1	-0.03	0.170	0.97	0.93	1.01
	**2**	**-0.06**	**0.037**	**0.94**	**0.88**	**0.99**
	*3*	*0.05*	*0.116*	*1.05*	*0.99*	*1.11*
Having a cat during pregnancy	1	-0.06	0.776	0.94	0.64	1.40
	**2**	**-0.49**	**0.071**	**0.62**	**0.36**	**1.04**
	*3*	*-0.02*	*0.949*	*0.98*	*0.57*	*1.69*
Having a dog during pregnancy	1	0.00	0.980	0.99	0.67	1.49
	**2**	**-0.55**	**0.050**	**0.58**	**0.33**	**1.00**
	*3*	*-0.25*	*0.401*	*0.78*	*0.43*	*1.40*
ETS-2	1	0.52	0.130	1.67	0.86	3.28
	**2**	**0.25**	**0.573**	**1.29**	**0.54**	**3.08**
	*3*	*-0.76*	*0.245*	*0.47*	*0.13*	*1.68*
CCG/CCG	1	-0.06	0.849	0.95	0.54	1.67
	**2**	**-0.57**	**0.163**	**0.57**	**0.25**	**1.26**
	*3*	*0.02*	*0.945*	*1.02*	*0.52*	*2.01*
ETS-2 × CCG/CCG	1	¥ -0.35	0.491	0.66	0.28	1.54
	**2**	¥**1.52**	**0.014**	**2.59**	**1.04**	**6.44**
	*3*	¥*0.70*	*0.409*	*2.06*	*0.45*	*9.33*
ETS-1	1	0.42	0.161	1.52	0.85	2.74
	**2**	**0.40**	**0.289**	**1.49**	**0.71**	**3.09**
	*3*	*0.16*	*0.687*	*1.17*	*0.54*	*2.52*
ETS-1 × CCG/CCG ¥	1	¥ 0.25	0.566	1.21	0.65	2.26
	**2**	¥ **0.48**	**0.410**	**0.92**	**0.40**	**2.07**
	*3*	¥ *-0.45*	*0.459*	*0.65*	*0.24*	*1.76*

ETS-0, mothers did not smoke during pregnancy and children not exposed to household ETS; ETS-1, mothers did not smoke during pregnancy, but children were exposed to household ETS; ETS-2, mothers smoked during pregnancy and children were exposed to household ETS.

† The most common haplotype pair, inferred from genotype data of five single nucleotide polymorphisms of interleukin 13 gene, rs1800925, rs2066960 and rs20541, using PHASE 2.0.2 software [34].

‡ correspond to a specific wheeze phenotype: Function 1: *early-transient *(n = 190), 2: *early-onset persistent *(n = 105), 3: *late-onset *(n = 70). The reference was the group who never report wheeze up to age 10 (n = 426).

× Denotes an interaction.

¥ For interaction terms, GLIMMIX estimates the Odds Ratio (95% CI) of the combined effect based on the parameter estimate of the main and the interaction effect. For the interaction term, we present the Odds Ratio for two combined effects, but not the statistical significant estimate of the individual interaction term. For example for *early-onset persistent *wheeze (function 2), the OR for children with the CCG/CCG genotype who had mothers who smoked during pregnancy is exponential (1.5223 + [-0.57]) = 2.59. The OR for maternal smoking during pregnancy (ETS-2 = 1) in children having the CCG/CCG genotype is exponential (-0.2512 + 1.5223) = 5.89 (95% CI: 2.47, 14.1). The difference in the combined effects results from different reference groups (no smoking during pregnancy and not the CCG/CCG genotype, respectively).

To determine the effect of minor haplotype pairs (frequency = 0.087, table 3), the analyses were repeated by excluding this group and results did not change significantly (data not shown).

When analyzing asthma, 68 subjects had persistent asthma. The control group were 533 subjects who did not have any asthma diagnosis during the first decade of life. When analyzing asthma instead of wheezing, the interaction between ETS-2 and *IL13 *haplotype pairs was statistically significant (p = 0.03) for persistent asthma as it was for persistent wheeze. Children with a CCG/CCG haplotype pair had an OR of 5.57 (95% CI 2.13 to 14.63, p = 0.0005) for ETS-2 on persistent asthma. For subjects with haplotype pairs other than CCG/CCG, the OR was 1.32 (95% CI 0.57 to 3.04, p = 0.587).

Among confounders, the presence of a household cat or dog during pregnancy was protective and male sex was a risk factor for *early-onset persistent *wheeze (table 5).

## Discussion

This study investigated the combined effect of exposure to tobacco smoke and haplotype pairs of the *IL13 *gene on wheeze phenotypes in the first decade of life using the data from the Isle of Wight birth cohort. Maternal smoking during pregnancy was associated with *early-transient *and *early-onset persistent *wheeze. No independent effect for the *IL13 *gene was detected. However, common variant of *IL13 *gene polymorphisms were observed to increase the adverse effect of maternal smoking during pregnancy on *early-onset persistent *wheeze, the phenotype with the highest morbidity [23]. A similar association was observed for the persistent asthma phenotype. Tobacco smoke exposure after pregnancy did not modify the association of *IL13 *and wheezing nor asthma.

In this study, the information was available from a subset of children who were followed up at age 10 and who agreed to provide blood for genotyping. Although these children appeared to have slightly (~3%) more wheeze and less exposure to tobacco smoke in comparison to all children who were followed up at age 10, these differences were not significant. The presence of a selection bias could result in a violation of the Hardy-Weinberg equilibrium and different allele frequencies than other Caucasian populations [29,38]. The latter scenario was not present, hence, a selection bias is unlikely.

The wheeze phenotypes were specified at age 10 based on longitudinal records from ages 1, 2, 4 and 10. This strengthens the study as we used longitudinal wheeze phenotypes instead of an outcome measured at a single cross section. To avoid recall bias, the analysis was restricted to children who were seen prospectively with information at all study visits [23]. Previously, we have shown that a preceding diagnosis of asthma was less likely to produce biased reports in later follow-ups [24].

Information on individual SNPs was used and the most likely pairs of haplotypes were estimated from genotype data. We demonstrated a significant association between exposure to maternal smoking during pregnancy and *early-onset persistent *wheeze when children had the common variant for each individual SNP as well as haplotype pairs.

One motivation for using haplotype pairs was the consistent pattern observed for the combined effect of ETS-2 and all markers (individual SNPs and haplotype pairs) on *early-onset persistent *wheeze (Figure 2). Additionally, it has been suggested, specifically for *IL13 *[31], that haplotype analysis could confer more information than individual marker analysis [39,40]. Haplotype pair analysis may misclassify genotypes when parents' genetic information is not available (ambiguous phase). However, the probability of having CCG/CCG, the major haplotype pair, was 1.00 in 429 out of 435 children with this genotype (the probability for the other 6 children was 0.89). The distribution of the data, with respect to minor haplotypes, did not allow testing for their interactions with tobacco smoke exposure. However, when children homozygous for minor haplotypes (frequency = 0.071, table 3) were removed from the analysis, the results did not change substantially.

Previous studies have shown an increased risk of asthma in children who were exposed to tobacco during pregnancy [3]. In a prior examination of this cohort, a possible association between exposures during pregnancy and *early-onset persistent *wheeze was suggested [37]. Additionally, several studies have suggested a gene-environment interaction for the effect of tobacco smoke exposure and asthma phenotypes [13,14,16]. Specifically, in two genome-wide screens, Colilla et al and Meyers et al reported that exposure to tobacco modifies the linkage between 5q, the region containing *IL13*, and asthma phenotypes [13,14]. The current study demonstrates a scenario in which a gene modifies the effect of tobacco smoke exposure during pregnancy but not after, thus, time of the exposure may be of critical importance in gene-environment interaction studies. The finding of an interaction between *IL13 *and ETS in the present study suggests that negative reports for the effect of a candidate gene for *IL13 *and asthma [20,21], could be explained by a failure to take into account environmental exposures. It is therefore of utmost importance, for genetic studies, to describe environmental exposures in the target population.

Most of the previous studies, including a report on the Isle of Wight cohort [41], suggest that the minor alleles of *IL13 *SNPs rs20541 (R130Q) and rs1800925 (-1112C/T) are associated with increased adverse effects [10,17-19,42]. In the current study we observed that children who had the common genotype for *IL13 *polymorphisms (haplotype pairs or SNP rs20541) have increased risk of *early onset persistent wheeze *and persistent childhood asthma in relation to maternal smoking during pregnancy.

Our finding for common *IL13 *variants to be a risk factor when mothers smoked during pregnancy is unexpected. Nevertheless, the observed association is statistically significant and cannot be explained by chance (p = 0.014). Additionally, we see a constant pattern for the effect of smoking across the SNPs and their haplotypes. This latter observation also suggests that it is less likely that the findings are due to a random effect. An unexpected finding for the effect of SNP rs20541 alleles on persistent wheeze is not scientifically untenable as the interplay between genes and environment is complex. Similar discrepancy has been shown for CD14, which turned out to be an interesting gene-environment interaction [43].

It has been suggested that tobacco smoke increases IL-13 and there are some reports on the combined effects of *IL13 *polymorphisms or other genetic variants in the *IL13 *region on asthma-related outcomes [11-14,44].

Liu *et al*. reported a synergistic effect of smoking and *IL13 *promoter polymorphism on the level of serum IgE [44], and genome-wide analyses have suggested a gene-environment interaction for the effect of tobacco exposure on asthma [13,14]. Noakes and colleagues demonstrated that cord blood cells produce significantly higher levels of IL-13 in response to both house dust mite and ovalbumin when newborns are exposed to tobacco smoke during pregnancy [11]. This suggests that prenatal exposure to tobacco elicits immunological effects. In our analysis, the interaction between tobacco exposure and *IL13 *polymorphisms was present in those who were exposed during and after pregnancy, but was not evident for the group with the same polymorphisms who were exposed only after pregnancy. As there was no group of children that was exposed only during pregnancy, it is not possible to distinguish definitively the effect of tobacco exposure before and after pregnancy. However, considering both the relatively short time of pregnancy and the large difference of risk between the two exposed groups, we suggest that exposure to tobacco during pregnancy has more influence on asthma than tobacco exposure after pregnancy.

In summary, in a sub-sample of the Isle of Wight cohort, the combined effect of exposure to tobacco smoke during pregnancy and the common haplotype pair of the *IL13 *gene resulted in an increased relative risk of *early-onset persistent *wheeze and asthma. For tobacco smoke exposure later in childhood we did not observe this association. The *IL13 *gene did not pose a risk in its own right. These results demonstrate that the association between exposures to environmental risk factors, like tobacco smoke, can be modified by gene polymorphisms. Given that there are various patterns and prevalences of exposure to tobacco smoke in different populations, this study suggests that negative reports of genetic association studies may be due to differences in environmental exposures. We propose that the next step in the investigation of the interaction between *IL13 *and exposure to tobacco smoke is to examine the influence of ETS exposure on *IL13 *expression.

## Authors' contributions

AS and WK designed the current study and conducted the analyses. SHA established the birth cohort and performed the clinical study. SHA and RK performed wheezing phenotypes analysis. MH aided on statistical analysis. SE performed the genetic analysis. All authors contributed in writing the manuscript and approved the final version.
